# Developing poly(ethylene glycol)-*b*-poly(β-hydroxybutyrate)-based self-assembling prodrug for the management of cisplatin-induced acute kidney injury

**DOI:** 10.1080/14686996.2024.2382084

**Published:** 2024-08-13

**Authors:** Duc Tri Bui, Yukio Nagasaki

**Affiliations:** a Degree Program of Pure and Applied Sciences, Graduate School of Science and Technology, University of Tsukuba, Ibaraki, Japan; b Department of Materials Science, Faculty of Pure and Applied Sciences, University of Tsukuba, Ibaraki, Japan; c Master’s School of Medical Sciences, Graduate School of Comprehensive Human Sciences, University of Tsukuba, Ibaraki, Japan; d Center for Research in Radiation and Earth System Science (CRiES), University of Tsukuba, Ibaraki, Japan; e Department of Chemistry, Graduate School of Science, The University of Tokyo, Tokyo, Japan; f High-Value Biomaterials Research and Commercialization Center (HBRCC), National Taipei University of Technology, Taipei, Taiwan

**Keywords:** Acute kidney injury, β-hydroxybutyrate, poly(ethylene glycol)-*b*-poly(β-hydroxybutyrate), nanoparticles, ketone bodies

## Abstract

Although β-hydroxybutyrate (BHB), one of the endogenous body ketones, possesses high bioactivities, it is rapidly consumed, metabolized, and eliminated from the body. In this study, we designed new self-assembling nanoparticles that sustainably released BHB to improve bioavailability and evaluated their efficacy in *in vivo* experiments using rodent animal models. Since poly(β-hydroxybutyrate) [poly(BHB)] is regarded as a polymeric prodrug that is hydrolyzed by endogenous enzymes and releases BHB in a sustained manner, our idea was to engineer hydrophobic poly(BHB) in one of the segments in the amphiphilic block copolymer, of which self-assembles in water to form nanoparticles of tens of nanometers in size (abbreviated as Nano^BHB^). Here, methoxy-poly(ethylene glycol) was employed as the hydrophilic segment of the block copolymer to stabilize the nanoparticles in aqueous environments, thus enabling Nano^BHB^s to be administrable both orally and through injection. Experimental results showed that Nano^BHB^ has low toxicity and releases free BHB for an extended period *in vitro* and *in vivo*. Moreover, Nano^BHB^ exhibits superior nephroprotective effects in cisplatin-induced acute kidney injury mouse models compared to low-molecular-weight (LMW) sodium BHB, suggesting the potential of Nano^BHB^ as a sustainable release formulation to supply BHB for medicinal applications.

## IMPACT STATEMENT

We engineered self-assembling poly(ethylene glycol)-*b*-poly(D,L-β-hydroxybutyrate) nanoparticles as a prodrug for continuous release of β-hydroxybutyrate, inducing bioactivity as a ketone body and providing nephroprotective properties against acute kidney injuries

## GRAPHICAL ABSTRACT

**Figure:**
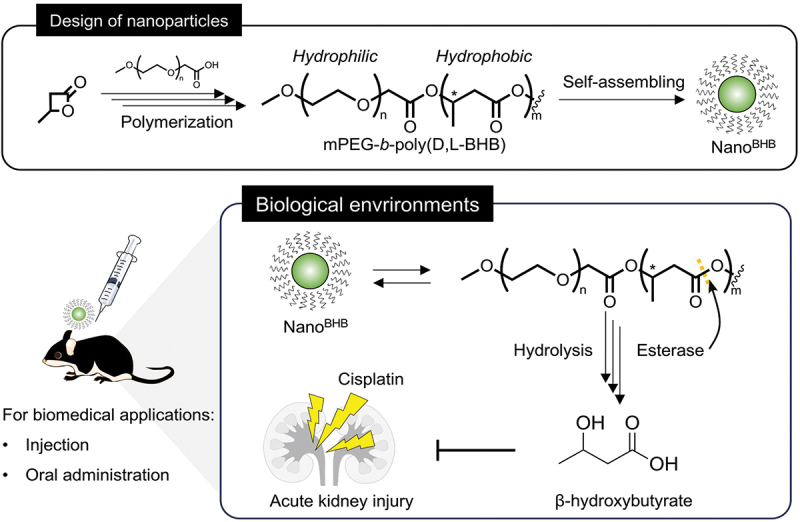

## 1. Introduction

Acute kidney injury (AKI) is a severe clinical complication associated with elevated mortality in hospitalized patients [1]. Given the kidney’s crucial role in maintaining homeostasis and eliminating toxins from the body, the organ is vulnerable to exogenous poisons and several medical interventions such as chemotherapy [2]. As a representative of anti-cancer drugs, cisplatin accumulates in the kidneys and causes damage with a morbidity of 20–30% of patients progressing to acute kidney injury after cisplatin therapy [3,4]. Reportedly, β-hydroxybutyrate (BHB), an endogenous ketone produced via β-oxidation pathways in livers, can alleviate aging progression [5] and disease pathology via regulation of gene transcription and inflammation response [6–11]. Mikami et al. reported the effects of BHB to attenuate cisplatin’s toxicity *in vitro* on human renal cortical epithelial cells by regulating the activity of endogenous histone deacetylase (HDAC) [12]. Following that, Luo et al. demonstrated *in vivo* that a one-shot injection of BHB just after administration of cisplatin protected mice against cisplatin-induced AKI, in addition to down-regulating the expression and activity of NLRP3 inflammasome in kidneys of cisplatin-injected mice [13]. In another model, Tajima et al. also reported the nephroprotective effects of continuous infusion of BHB salt in renal ischemia-reperfusion through HDAC inhibitory effects [14], suggesting that BHB has promising nephroprotective effects for managing AKI.

Several strategies have been introduced to boost BHB levels, including fasting, exercise, diet, or supply of exogenous BHB supplements [5]. While diet and lifestyle methods require rigorous obligation and efforts to reach the target BHB levels, using exogenous BHB sources would be more convenient and suitable for most people. BHB supplements are commercially available in the form of sodium, calcium, and magnesium salts. However, BHB supplied from conventional products is rapidly consumed, metabolized, and excreted due to its small molecular weight and hydrophilic nature [15]. This results in a lower bioavailability of BHB in target organs and hinders its therapeutic efficacy [16].

It is reported that poly(β-hydroxybutyrate) polymer, abbreviated as poly(BHB), accumulates as granules in the cytoplasm of marine microorganisms [17]. As a polyester, poly(BHB) is a possible target of endogenous enzymes, which digest ester bonding to release BHB as ketone bodies [18–20], thereby exerting beneficial effects on a living body. Hence, a new strategy using poly(BHB) as a source of BHB has been recently proposed [5]. For example, a fed diet mixed with bacteria-produced poly(BHB) showed anti-tumor effects in rat models with colorectal cancers [21] and anti-inflammatory effects in inflammatory bowel disease mouse models [22]. However, poly(BHB) is water-insoluble and can exist in either a brittle solid or elastic form [23]. These characteristics hinder poly(BHB)’s potential in medicinal applications as the physiological environment favors dissolvable or well-dispersed formulations in aqueous conditions. For instance, it is impossible to inject insoluble material into the body due to the risk of clogging blood vessels.

Our previous studies have successfully addressed the aforementioned challenges of various hydrophobic polymers for medicinal applications by employing the hydrophilicity of methoxy-poly(ethylene glycol) (mPEG) to form and stabilize core-shell nanoparticles. These nanoparticles gradually liberate covalently bonded low-molecular-weight bioactive compounds such as short-chain fatty acids and amino acids [24–27]. For example, Shashni et al. synthesized di-block copolymers of poly(vinyl butyrate)s and mPEG to prepare administrable poly(vinyl butyrate)-based nanoparticles that enable the release of butyric acid and have demonstrated therapeutic efficacy in various disease models such as diabetes and nonalcoholic fatty liver disease [26,28].

**Figure 1.**
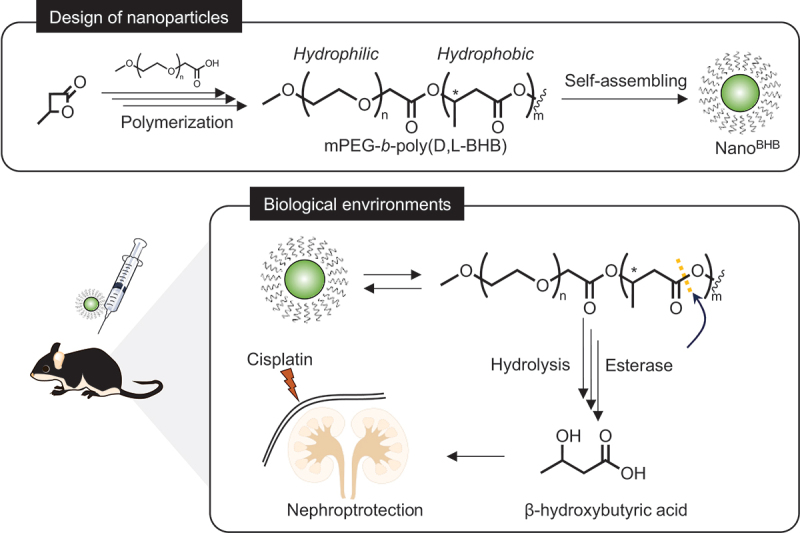
Design of poly(β-hydroxybutyrate)-based nanoparticles and strategy to protect kidneys.

Using this strategy, this study proposes an improved formulation of poly(BHB) for drug administration by synthesizing the methoxy-poly(ethylene glycol)-*b*-poly(β-hydroxybutyrate) (mPEG-*b*-poly(BHB)) di-block copolymer and preparing stable nanoparticles in aqueous environments. In nature, poly(BHB) homopolymers are produced by bacterial biosynthesis in microorganism biomass. Alternatively, it is chemically synthesizable by the ring-opening polymerization of β-butyrolactone [29,30]. The bacteria-produced polymer exists in isotactic poly(D-BHB) form with strong crystallinity; meanwhile, chemically synthesized polymers can be designed with diverse configurations, dependent on the stereoisomer ratio of the monomer used [23,31]. For example, atactic poly(D,L-BHB) forms with lower crystallinity can be synthesized in a simple manner from commercially available racemic [D,L]-β-butyrolactone [19]. Since the high crystallization tendency of natural isotactic poly(D-BHB) decreases the required mobility of amphiphilic polymer for the self-assembling process, it is challenging to prepare stable and small-size nanoparticles from the di-block copolymer of natural poly(D-BHB) [32]. Hence, we employed the self-assembling nanoparticle using di-block copolymers with synthetic atactic poly(D,L-BHB), synthesized from the ring-opening polymerization of [D,L]-β-butyrolactone in the presence of mPEG-macroinitiators. The as-designed nanoparticles, namely Nano^BHB^, showed stability in their nanometer form and can be projected to be endogenously administrable by different routes (including oral and injection). With the biodegradability to release free BHB sustainably through enzymatic hydrolysis, Nano^BHB^ would improve the bioavailability of BHB at target organs and ameliorate BHB’s efficacy of treatments in cisplatin-induced AKI mouse models (Figure 1).

## 2. Material and methodology

### 2.1. Materials and animals

#### 2.1.1. Chemicals

Monomethoxy-poly(ethylene glycol) (MW = 2000, mPEG-OH) was purchased from Fluka, Japan. Ethyl bromoacetate, 18-crown-6 ether, and [D,L]-β-butyrolactone were purchased from Tokyo Chemical Industry Co., Tokyo, Japan (TCI). Butyllithium (1.6 M in hexane) and sodium β-hydroxybutyrate were purchased from Sigma-Aldrich. 2-Propanol, methanol, hexane, dichloromethane, sodium hydroxide, hydrochloric acid, and N,N-dimethylformamide (DMF) were purchased from FUJIFILM Wako Pure Chemical Co., Osaka, Japan. Super-dehydrated tetrahydrofuran (THF, purchased from Kanto Chemical Co., Japan) was stored and used via a solvent purification system (GlassContour, Nikko-Hansen, Japan). Potassium naphthalene (0.9 M in hexane; KNaph) was prepared in our laboratory and stored at −30°C.

#### 2.1.2. Animals

Male C57BL/6J mice (6–8 w old) and male ICR mice (6 w old) were purchased from Jackson Laboratory, Japan. Animal experiments were conducted at the Laboratory Animal Resource Center, University of Tsukuba, Japan. Mice were housed at standard conditions with temperature (23.5 ± 2.5 °C), humidity (52.5 ± 12.5%), and light-dark cycle (lights on from 7:00–19:00). The design of animal experiments was approved with the plan #23–321 according to the Regulation for Animal Experiments at the University of Tsukuba.

### 2.2. Synthesis of mPEG-b-poly(D,L-BHB)

#### 2.2.1. Synthesis of monomethoxy-poly(ethylene glycol) carboxylic acid

Monomethoxy-poly(ethylene glycol) with a carboxylic acid group at the chain end (mPEG-COOH) was synthesized from monomethoxy-poly(ethylene glycol) (mPEG-OH, MW = 2000) as described in a previous study [25]. Briefly, mPEG-OH (20 g, 10 mmol) was placed in a round-bottomed flask, purged with N_2_, and dissolved with super-dehydrated THF (50 mL). Butyllithium (10 mL, 16 mmol) and ethyl bromoacetate (3.6 mL, 30 mmol) were added in this order and stirred for 1 day at room temperature. The mixture was poured into 500 mL cold 2-propanol (−30 °C), and the precipitate was collected by centrifugation. After completely being dried under reduced pressure, 18 g of the obtained white powder was placed in another round-bottomed flask, dissolved in MilliQ water (50 mL), and hydrolyzed using NaOH 1.5 M (10 mL, 15 mmol). After 1 day with stirring, pH was adjusted to 1–2 by HCl 1 M aqueous solution. The target product was extracted with dichloromethane (60 mL) three times with the collection of organic phases. Dichloromethane was evaporated, and the product was purified through the precipitations in cold 2-propanol three times. The mPEG-COOH was collected as a white powder after drying under reduced pressure (16.0 g, 80% yield). ^1^H-NMR (400 MHz, CDCl_3_, 25 °C): δ 3.34 (3 H, s, –OC*H*_3_), 3.80–3.40 (–OC*H*_*2*_C*H*_*2*_ –), 4.11 (2 H, s, –OC*H*_*2*_COO–).

#### 2.2.2. Synthesis of mPEG-b-poly(D,L-BHB)

mPEG-COOH (2.0 g, 1 mmol) was placed in a round-bottomed flask, purged with N_2,_ and dissolved with super-dehydrated THF (15 mL). KNaph 0.9 M/hexane (1.5 mL, 1.35 mmol) was added dropwise, followed by the addition of 18-crown-6 ether 1 M/THF (2 mL, 2 mmol) and [D,L]-β-butyrolactone (4 mL, 49 mmol), sequentially. After reacting for 2 days, ethyl bromoacetate (4 mL, 33 mmol) was added to stop the reaction. The product was purified through precipitation in a mixture of 2-propanol/hexane (20/80) three times and vacuum-dried to obtain mPEG-*b*-poly(BHB) copolymer. ^1^H-NMR (400 MHz, CDCl_3_, 25 °C): δ 1.26–1.31 (m, –OCH(C*H*_*3*_)CH_2_CO – of poly(BHB) block), 2.40–2.68 (–m, –OCH(CH_3_)C*H*_*2*_CO – of poly(BHB) block), 3.34 (s, –OC*H*_3_ of mPEG block), 3.80–3.40 (m, –OC*H*_*2*_C*H*_*2*_– of mPEG block), 4.11 (s, –OC*H*_*2*_COO – of mPEG block), 5.18–5.40 (–m, –OC*H*(CH_3_)CH_2_CO – of poly(BHB) block). GPC(DMF): M_w_(GPC) = 5100, M_n_(GPC) = 4900, M_w_/M_n_ = 1.04.

### 2.3. Preparation of self-assembling nanoparticles (Nano^BHB^)

MilliQ water (40 mL) was added dropwise in a DMF solution of mPEG-*b*-poly(BHB) (140 mg/mL, 40 mL) under vigorous stirring. The mixture was dialyzed against 2 L of MiliQ Water in a 3.5 kDa RC dialysis membrane for 3 days (water was replaced twice a day). Finally, the nanoparticle solution was filtered by a 0.2 µm-pore size filter to remove any aggregation and bacteria that might be contained in the sample, resulting in the final product of Nano^BHB^. For injection purposes, nanoparticles in saline were prepared by mixing nanoparticles solution with 9% of NaCl (9:1). All the nanoparticles were stored at 4°C for further use.

### 2.4. Evaluating the release of BHB from Nano^BHB^

*In vitro enzymatic hydrolysis*: A mixture containing either Nano^BHB^ (19 mg/mL, 1 mL) or poly(D,L-BHB) (19 mg/mL, 1 mL), with porcine esterase (1 mg/mL, 1 mL) and MilliQ water (2 mL) was incubated at 37°C. After designated incubation times (i.e. *t =* 5 min, 2 h, 6 h, 12 h, 24 h, 48 h), 100 µL of the mixture was collected (*n* = 3) and immediately frozen in liquid N_2_. After the sample collection at the timepoint of *t* = 48 h, KOH 1 M (1 mL, 1 mmol) was added and incubated for 4 days to completely proceed with the hydrolysis, at which the timepoint was considered as *t* = ∞. Additionally, the levels of BHB in the mixture containing Nano^BHB^ only, poly(D,L-BHB) only, and esterase only were measured at 5 min and 48 h. The accumulated release of BHB was measured using an LC-MS/MS system. Here, 100 μL of the sample was mixed with 900 μL methanol (MeOH), followed by centrifugation to remove any aggregation. 10 μL filtered supernatant was injected into the LC-MS/MS system for BHB detection. The accumulated BHB was calculated as BHB (%) = [BHB]_t_ ×100%/[BHB]_t=∞_.

*In vivo release by intraperitoneal (i.p.) injection in mice*: ICR male mice (6 weeks old) were administered with 0.4 mL Nano^BHB^ (30 mg/mL) or Sodium β-hydroxybutyrate (29 mg/mL) by i.p. injection. After designated time points (i.e. 10 min, 20 min, 45 min, 1.5 h, 3 h, 6 h, 12 h), mice were sacrificed to collect plasma (*n* = 3). Plasma samples were directly frozen in liquid N_2_ and stored at −80°C for further analysis. The levels of BHB in plasma samples were measured using an LC-MS/MS system. Here, 50 μL plasma was mixed with 450 μL MeOH, followed by centrifugation to remove protein. 10 μL of the filtered supernatant was injected into the LC-MS/MS system for BHB detection.

### 2.5. Pharmacokinetic study by s.c. injection

ICR male mice (6 weeks old) were administered with 0.4 mL of either Nano^BHB^ or Sodium β-hydroxybutyrate at the dose of ca. 345 mg-BHB/kg by s.c. injection. After designated time points (i.e. 20 min, 45 min, 1.5 h, 3 h, 6 h, 12 h), mice were sacrificed to collect plasma, kidney, and liver. Plasma and tissue samples were directly frozen in liquid N_2_ and stored at −80°C for further analysis. The levels of BHB in plasma, kidney, and liver were measured via LC-MS/MS. In the case of plasma, 50 μL was mixed with 450 μL MeOH, followed by centrifugation to remove proteins. 10 μL of the filtered supernatant was injected into the LC-MS/MS system for BHB detection. In the case of the liver and kidney, about 200 mg of wet tissues were homogenized with 1 mL MiliQ water, followed by centrifugation to collect the supernatant. Then, 50 μL supernatant was mixed with 450 μL MeOH, followed by centrifugation to remove proteins. 10 μL filtered supernatant was injected into the LC-MS/MS system for BHB detection.

### 2.6. BHB detection using an LC-MS/MS system

The amount of BHB in the samples was measured using an LC-MS/MS system (API 2000, AB SCIEX, Canada). For the detection of BHB, the LC condition (eluent: MeOH 50%; flow rate: 0.1 mL/min; and the column: TSKgel ODS-100Z or TSKgel ODS-100 V) and the MS/MS condition (negative mode, fragment: 103.0/59.0) were applied with the measuring time of 7 min in each sample.

### 2.7. Protection against cisplatin-induced acute kidney injury

C57BL/6J male mice (8 weeks old) were divided into four groups with different treatments. Acute kidney injuries were induced by i.p. injection of cisplatin (20 mg/kg). Six hours before the exposure to cisplatin, mice were pre-treated using either saline (*n* = 11), sodium β-hydroxybutyrate (ca. 250 mg-BHB/kg, *n* = 10), or Nano^BHB^ (ca. 250 mg-BHB/kg, *n* = 10) via s.c. injection. The healthy control group (*n* = 8) was injected with saline instead of cisplatin. After 72 h from the cisplatin challenge, mice were sacrificed to collect plasma and kidneys. Kidney functions were evaluated by measuring the levels of Blood Urea Nitrogen (BUN), Creatinine (CRE), and Aspartate transaminase (AST). In all cisplatin-injected groups, the two mice with the most scattered creatinine levels were excluded from the data analysis.

### 2.8. Histological analysis

Paraffin-embedded kidney tissues collected from cisplatin-induced AKI experiments were stained with Periodic acid–Schiff (PAS). Injury areas were identified as the protein areas casted with a uniform pink color in the PAS staining section and were quantitatively measured by ImageJ.

### 2.9. Blood and plasma biochemistry analysis

Total blood count was measured using a hematology analyzer (Celltac α MEK6458; Nihon Kohden, Japan). Plasma biochemistry analysis was measured via dry chemistry using an automatic clinical chemistry analyzer (DRI-CHEM 7000 V; FUJIFILM, Japan).

### 2.10. Toxicity study

#### 2.10.1. In vitro cytotoxicity

Bovine Aorta Endothelial Cells (BAEC purchased from RIKEN, Tsukuba, Japan) pre-seeded on a 96-well plate were incubated with either Nano^BHB^ or BHB at different concentrations ranging from 0 to 25.4 mM of BHB content in culture media. After 24 h, the media were discarded, followed by adding 100 µL of culture media containing 10 µL of WST agent and incubating for 4 h. In the final step, the cell viability was measured based on the ratio of optical signals at the 450 nm wavelength of each well to that of the non-treated group.

#### 2.10.2. In vivo toxicity experiment

C57BL/6J male mice (8 weeks old) were divided into two groups and administered an s.c. injection of either Nano^BHB^ (ca. 250 mg-BHB/kg, *n* = 5) or saline (*n* = 5). After 3 days from the injection, mice were sacrificed to collect total blood, plasma, and major organs (kidney, liver, and spleen). Kidney functions were evaluated by measuring the levels of blood urea nitrogen (BUN), creatinine (CRE), aspartate transaminase (AST), and alanine transaminase (ALT).

#### 2.11. Statistical analysis

Statistical analysis was performed using GraphPad Prism 8 software. The differences among the means were determined using Student’s t-test (for comparison between two groups) and one-way ANOVA followed by Turkey’s multiple comparison tests (for comparisons among more than two groups). Data in figures are displayed as mean ± standard deviation (SD), with *p*-value < 0.05 considered as the significant difference (*p ≤ 0.05, **p ≤ 0.01, ***p ≤ 0.001, ****p ≤ 0.0001).

## 3. Results and discussion

### 3.1. Synthesis and characterization of mPEG-b-poly(D,L-BHB)

**Figure 2.**
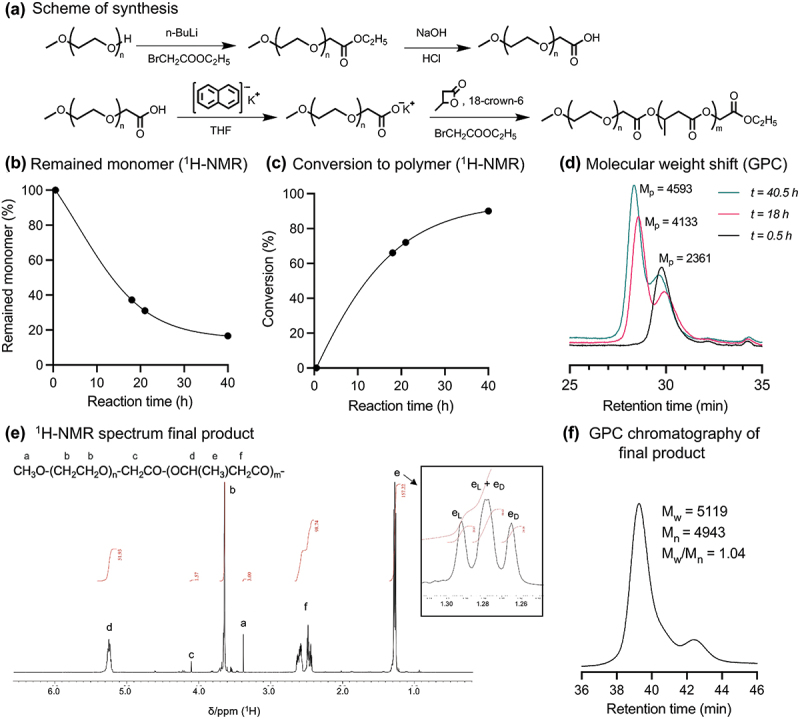
Synthesis and characterization of mPEG-*b*-poly(BHB).

The atactic mPEG-*b*-poly(D,L-BHB) di-block copolymers were synthesized through a ring-opening polymerization process of racemic [D,L]-β-butyrolactone, initiated with mPEG-carboxylate anion (mPEG-COO^−^) (Figure 2(a)). Initially, the hydroxyl end group of mPEG-OH was converted to a carboxylate ester group via a Williamson ether synthesis reaction. The ester bond was then hydrolyzed under alkaline conditions, and the pH of the mixture was adjusted to obtain mPEG with a carboxylic acid end group, mPEG-COOH, of which the structure was confirmed by ^1^H-NMR spectrum (Figure S1). In a one-pot reaction, mPEG-COOH underwent deprotonation using the KNaph reagent, forming potassium carboxylate in THF. 18-Crown-6 ether was utilized to trap the potassium ion, thereby exposing the naked carboxylate anion for ring-opening polymerization of β-butyrolactone [29,30]. The polymerization progress was monitored using ^1^H-NMR, where the signal of β-butyrolactone decreased over reaction times, as demonstrated in Figure 2(b). After 40.5 h, the conversion of the monomer reached approximately 80% (Figure 2(c)); thus, the reaction time was fixed at 2 days in a standard protocol. In addition, gel permeation chromatography (GPC) demonstrated the shift of initial peak to higher molecular weight regions over time (Figure 2(d)), indicating the polymerization occurring from the end of PEG-macroinitiators and the successful formation of block copolymers. The small peak observed on the right side of the chromatograms is the signal of unreacted PEG in the reaction medium during polymerization.

(a) Scheme of synthesis. (b) The calculated amount of remaining monomer and (c) the conversion to polymer in the reaction mixture over reaction time monitored by ^1^H-NMR measurement. (d) The GPC measurement in THF of reaction mixtures collected at different reaction times. (e) ^1^H-NMR spectrum and (f) GPC measurement in DMF of mPEG-*b*-poly(D,L-BHB) after purification.

After 2 days of reaction, the polymerization was stopped by adding an excess amount of ethyl bromoacetate. The final products were purified and characterized in terms of chemical structures and molecular weights. The ^1^H-NMR spectrum of the product showed new high-intensity peaks at δ 1.26–1.31, δ 2.40–2.68, and δ 5.18–5.40 assignable to the signals of poly(D,L-BHB) as peak e, f, d, respectively, as shown in Figure 2(e), accompanied by the proton peaks of methoxy-poly(ethylene glycol) segment (peak a, b, c). This indicates the presence of both mPEG and poly(D,L-BHB) segments with the estimated degree of polymerization (DP) of 50 units for the poly(D,L-BHB) segment.

As racemic [D,L]-β-butyrolactone was employed for the synthesis of mPEG-*b*-poly(D,L-BHB) di-block copolymers, the random tacticity was confirmed by the multiplet peak at δ 1.25–1.30 due to the splitting of D- and L-configuration of monomer units (peak e, Figure 2(e)). Similarly, the splitting of D- and L-stereoconfiguration was observed in the ^13^C-NMR spectrum (Figure S2), confirming the acquisition of atactic poly(D,L-BHB) segment with a [D]/[L] ratio of approximately 1:1 as per our design. As mPEG with a molecular weight of 2000 Da was employed for the synthesis, the increased molecular weight of the final product (M_w_(GPC) = 5100 with M_w_/M_n_ = 1.04, Figure 2(f)) confirmed the obtainment of the mPEG-*b*-poly(BHB) di-block copolymers.

### 3.2. Preparation and characterization of self-assembling polymer micelle nanoparticles

**Figure 3.**
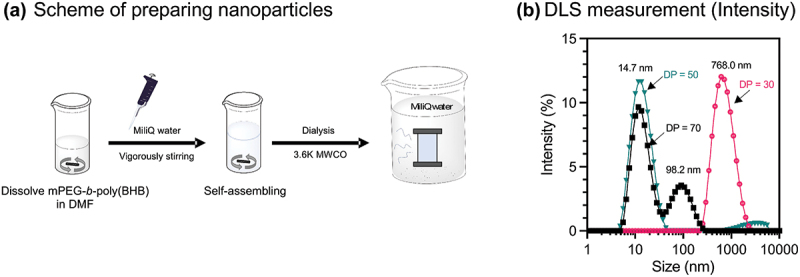
Preparation of nanoparticles and optimization of the design.

Nano^BHB^ was prepared using a simple dialysis method based on the self-assembly of amphiphilic polymers in water (Figure 3(a)). mPEG-*b*-poly(D,L-BHB) copolymers were dissolved in DMF, and MilliQ water was added dropwise during stirring to trigger the self-assembling formation of nanoparticles. DMF used was then removed by dialysis against water, yielding Nano^BHB^ nanoparticles. For optimization, we investigated the self-assembly of mPEG-*b*-poly(BHB) di-block copolymers with different DP of poly(BHB) segments. These were synthesized by changing the monomer/initiator ratio ([D,L]-β-butyrolactone versus mPEG-COO^−^) during synthesis, and their resulting characterizations are summarized in Table 1. Dynamic light scattering (DLS) measurements (Figure 3(b), S3) demonstrated that self-assembly of di-block copolymer with a short DP (DP = 30) resulted in large-size particles (d = 768.0 ± 367.2 nm). This is probably due to the insufficient coagulation force of the hydrophobic poly(D,L-BHB) segment, which leads to phase mixed aggregation. Meanwhile, copolymers with higher DP (DP = 50, 70) could produce small-size particles in the nanoscale (Figure 3(b), S3). Although the DLS measurement of DP = 70 showed dual peaks in size distribution within intensity, a single peak was observed in size distribution within volume (Figure S3). Since the scattering intensity is proportional to the ten to the sixth power of the magnitude of size magnitude [33], the intensity mode emphasizes more on larger aggregates, suggesting that a small number of aggregates are formed in the case of DP = 70 copolymers. Based on these results, an amphiphilic copolymer with 50–70 units of BHB is required to form stable nanoparticles.

(a) Scheme of nanoparticle preparation; (b) Hydrodynamic size of self-assembling particles from the di-blocks copolymer with different degrees of polymerization (DP) from DLS measurement.

**Table 1.** Summary of characterization of di-blocks copolymer with different DP.

DP	[β-butyrolactone]_0_/ [mPEG-COOH]_0_	M_n_ (^1^H-NMR)	M_w_ (GPC)	Hydrodynamic size (nm)
30	33	4580	4217	768.0 ± 367.2
50	66	6200	5285	14.7 ± 6.7
70	99	8106	5862	14.6 ± 6.7; 98.2 ± 44.7

**Figure 4.**
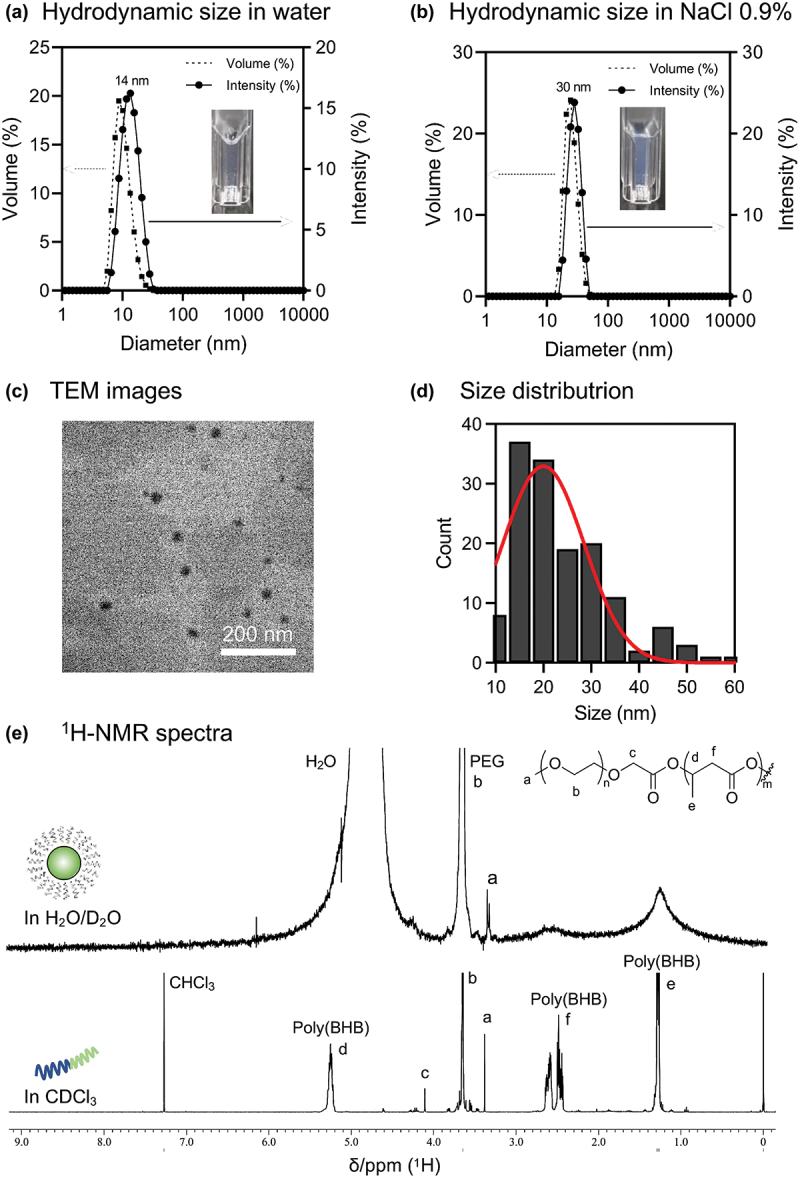
Characterization of Nano^BHB^.

In this study, the mPEG-*b*-poly(BHB) di-block copolymer with DP = 50 was chosen as the optimal design for the rest of the experiments. Although a trace amount of unreacted PEG remained in the sample (Figure 2(f)), clear nanosized self-assembling polymer micelles were observed after the dialysis. The nanoparticles, hereby denoted as Nano^BHB^s, were prepared in MilliQ water with small hydrodynamic diameters (*d*_*H*_ *=* 13.9 ± 4.4 nm) and a narrow polydispersity (DPI = 0.103) confirmed by DLS measurement (Figure 4(a)). Also, Nano^BHB^s in 0.9% NaCl solution are stable in nanometer forms with *d*_*H*_ *=* 29.6 ± 7.0 nm and PDI = 0.012 as depicted in Figure 4(b). These small sizes and uniformed dispersions make Nano^BHB^s suitable for both oral administration and injection route. In addition, the transmission electron microscopy (TEM) image with negative staining showed the presence of spherical nanoparticles with sizes ranging from 10 nm to 60 nm (Figure 4(c,d)), confirming the morphology of nanoparticles. We also conducted ^1^H-NMR measurements of Nano^BHB^ in aqueous media. As shown in Figure 4(e), the proton signals of poly(BHB) segments are nearly entirely diminished, while that of the mPEG block remained observable. These results indicate that the hydrophobic poly(BHB) collapsed into the nanoparticles’ solid core, leading to restricted mobility and prolonged T2 relaxation time in NMR measurement, resulting in the disappearance of its corresponding peaks. This is strong evidence that Nano^BHB^ forms a core-shell structure in aqueous media.

(a) Hydrodynamic size and inserted digital photo of Nano^BHB^ in MilliQ water; (b) Hydrodynamic size and inserted digital photo of Nano^BHB^ in NaCl 0.9%; (c) Representative of TEM images and (d) size distribution of Nano^BHB^ measured from TEM images. (e) ^1^H-NMR spectra of Nano^BHB^ solution in D_2_O and freeze-dried Nano^BHB^ in CDCl_3_.

### 3.3. Biodegradability of Nano^BHB^

**Figure 5.**
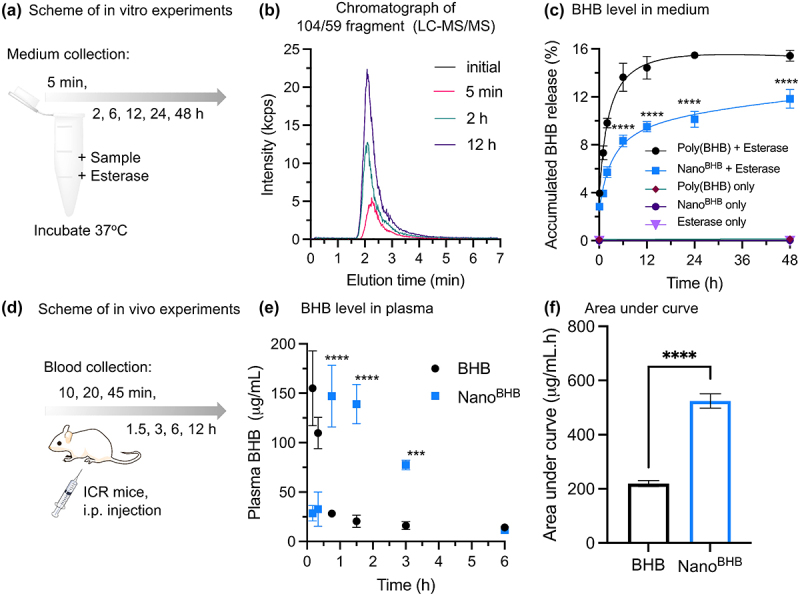
Biodegradability of Nano^BHB^.

Numerous studies have reported the biodegradation of natural poly(D-BHB) via the hydrolysis catalyzed by enzymes [18,34]. However, enzymatic biodegradation of atactic poly(D,L-BHB), especially by mammalian endogenous enzymes, is not yet widely reported. Therefore, the biodegradation of random atactic poly(D,L-BHB) under the effects of endogenous esterase was investigated *in vitro* and confirmed by the amount of free BHB released in incubation mediums. Here, the poly(D,L-BHB) homopolymers with M_n_(GPC) = 3453 were synthesized from [D,L]-β-butyrolactone in a reaction initiated by 4-phenylbutyric acid (Figure S4). This was then incubated with porcine esterase as described in Figure 5(a). The LC-MS/MS analysis of the liberated BHB from poly(D,L-BHB) showed that the BHB levels were remarkably increased over time with enzymes (Figure S5) and significantly higher than that without enzymes after 48 h (Figure 5(c)). The above results demonstrate that chemically synthesized poly(D,L-BHB) can also be enzymatically hydrolyzed like its natural poly(D-BHB). Since Nano^BHB^ is composed of random atactic poly(D,L-BHB) as a hydrophobic segment, an *in vitro* biodegradation experiment of Nano^BHB^ was similarly conducted and compared to that of poly(D,L-BHB) homopolymer. LC-MS/MS chromatography analysis demonstrated an increase in detected BHB within the mixture of Nano^BHB^ and esterase over time (Figure 5(b)). This suggests that Nano^BHB^ is degradable and releases BHB induced by the enzymatic hydrolysis similar to that of poly(D,L-BHB) homopolymer. To note, quantitative analysis showed that Nano^BHB^ released BHB slower but more steadily than homopolymer (Figure 5(c)), providing evidence for the protective effects of the peripheral mPEG layer in the core-shell structure of Nano^BHB^. Specifically, the mPEG layer hindered the direct interaction between poly(D,L-BHB) in the core and esterase enzyme, slowing down subsequent hydrolysis (thermodynamically stable condition of the polymer micelles) [35].

*In vitro experiments*: (a) Scheme of enzymatic degradation; (b) LC-MS/MS chromatograph with BHB detection of the mixture of Nano^BHB^ and esterase over incubation time; (c) Quantitative analysis of BHB levels in incubating medium over time (****p < 0.0001 vs. Poly(BHB)+Esterase group). *In vivo* experiments: (d) Scheme of BHB release profile in mouse models; (e) BHB levels in plasma after i.p. injection samples, measured by LC-MS/MS; (f) Area under the curve of BHB level in plasma. (Data are displayed as Mean±SD, *n* = 3, ***p < 0.001; ****p < 0.0001).

An animal experiment with mouse models was conducted to investigate the ability of Nano^BHB^ to release BHB *in vivo* (Figure 5(d)). Nano^BHB^ was i.p. injected into mice at a dose of 345 mg-BHB/kg, and blood was collected at designated time points to measure BHB level by LC-MS/MS measurement. From our observation, poly(D,L-BHB) homopolymers formed sticky aggregates in water (see the photo in Figure S6) and would clog injection needles; thus, the *in vivo* degradation of homopolymers was not investigated in this study. Instead, Nano^BHB^ was compared with sodium BHB as a conventional LMW counterpart. As shown in Figure 5(e), plasma BHB levels were immediately elevated (within 10 min) after i.p. injection of sodium BHB. However, it sharply decreased and returned to the background level within 1.5 h. In contrast, plasma BHB levels reached a maximum level at approximately 1 h after i.p. injection of Nano^BHB^ and remained at a significantly higher level for more than 3 h compared to that of the sodium BHB injection. This thus confirms the ability of Nano^BHB^ to supply BHB *in vivo* for an extended period of time. Additionally, the significantly higher area under the curve (AUC) of Nano^BHB^ compared to that of sodium BHB (Figure 5(f)) indicates that Nano^BHB^ is a plausible source of supplying sustained BHB and providing improved bioavailability of BHB in blood than conventional LMW compounds.

### 3.4. Safety of Nano^BHB^

**Figure 6.**
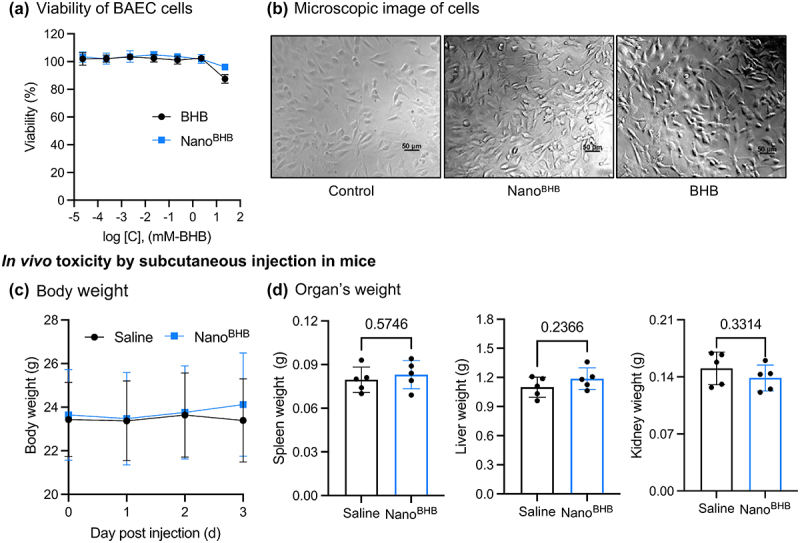
Safety of Nano^BHB^.

In order to evaluate the safety of Nano^BHB^, we investigated the cytotoxicity *in vitro* and organ damage *in vivo*. Cytotoxicity experiments were conducted with bovine aortic endothelial cells (BAEC) as a representative of normal cells. After 24 h co-incubation with Nano^BHB^ at various concentrations, BAEC did not exhibit remarkable changes in morphology and viability compared to that of the control non-treated group or sodium BHB-treated group (Figure 6(a,b)), suggesting that Nano^BHB^ with concentrations up to 23 mM-BHB (2.9 mg-polymer/mL) possesses low cytotoxicity to normal cells *in vitro*.

*In vitro cytotoxicity experiment with 24 h incubation with Nano*^*BHB*^: (a) Viability of bovine aortic endothelial cells (BAEC) (Mean ± SD, *n* = 4); (b) Representative microscopic images of BAEC cells after 24 h co-incubation (scale bar 50μm); *In vivo toxicity experiment by one-shot subcutaneous (s.c.) injection to mice*: (b) Body weight changes; (d) Spleen, liver, kidney’s weight after 3 days from the injection (Data are displayed as Mean ± SD, *n* = 5).

Later, Nano^BHB^ was subcutaneously (s.c.) injected into mice to investigate the toxicity of Nano^BHB^ *in vivo*. As shown in Figure 6(c), there was no significant difference in body weight between the Nano^BHB^- and saline-treated mice groups. Similarly, Nano^BHB^ did not induce changes in the weight of test mice spleens, liver, and kidney, as confirmed using the Student’s t-test (Figure 6(d)). Total blood count measurements revealed that the levels of white blood cells, red blood cells, hemoglobin, and platelets were not affected by the Nano^BHB^ treatment (Table 2). In addition, the liver and kidney’s functional damage biomarkers (alanine transaminase, aspartate transaminase, blood urea nitrogen, and creatinine) remained at similar levels in plasma when treated with either Nano^BHB^ or saline (Figure 6(e)). This suggests that Nano^BHB^ at the dose of 250 mg-BHB/kg did not have an adverse effect on metabolism and clearance function within healthy mice. Overall, Nano^BHB^ exhibited minimal cytotoxicity *in vitro* and demonstrated good tolerance *in vivo* after subcutaneous injection, providing evidence for the biocompatibility of Nano^BHB^ and further supporting its potential for medicinal applications.

**Table 2.** Total blood count and plasma biochemistry analysis from blood samples collected after 3 days from the injection.

	Saline	Nano^BHB^	*p*-value
White blood cell (10^2^/μL)	35.6 ± 5.4	35.6 ± 9.2	1.00
Red blood cell (10^4^/μL)	854.4 ± 38.1	826.6 ± 23.9	0.20
Hemoglobin (g/dL)	13.1 ± 0.5	12.9 ± 0.6	0.65
Platelet (10^4^/μL)	82.1 ± 1.5	78.5 ± 12.3	0.54
Alanine transaminase (U/L)	23.2 ± 3.3	25 ± 3.6	0.40
Aspartate transaminase (U/L)	53.4 ± 9.4	58.2 ± 11.7	0.50
Blood urea nitrogen (mg/dL)	29.7 ± 2.6	30.9 ± 2.1	0.45
Creatinine (mg/dL)	0.17 ± 0.01	0.22 ± 0.06	0.18

### 3.5. Assessment of nephroprotective effects in cisplatin-induced AKI models

**Figure 7.**
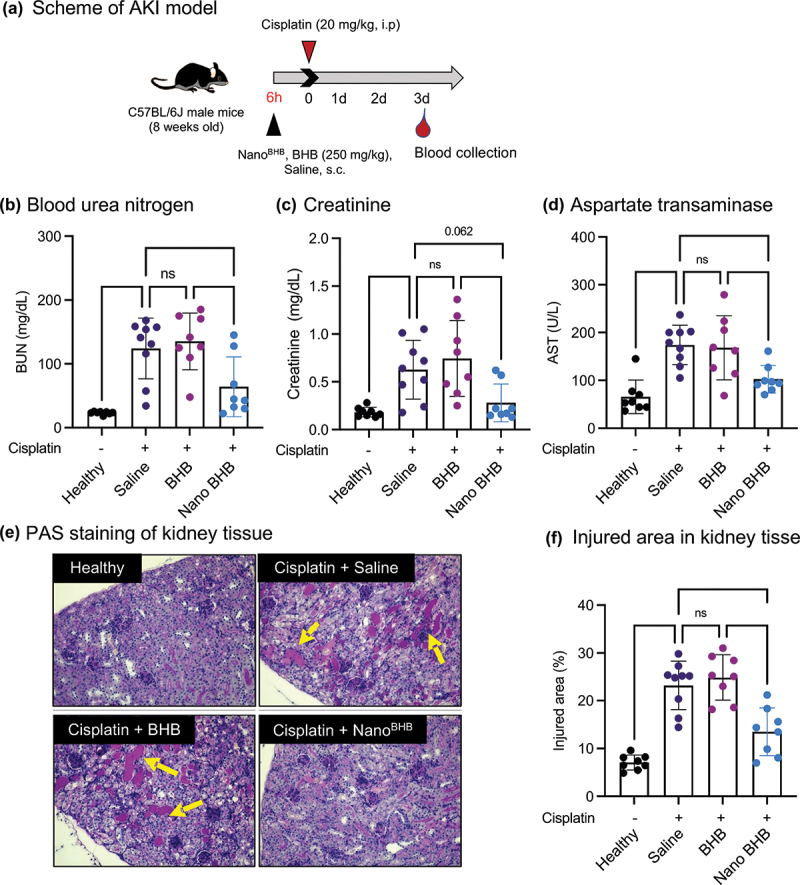
Nephroprotective effects of Nano^BHB^ in cisplatin-induced AKI models.

Mouse models of AKI induced by i.p. cisplatin injection were prepared to confirm the effect of Nano^BHB^. To avoid the large amount of liquid injected into mice’s peritoneum and the possible direct interaction with cisplatin, testing treatments (either saline, Nano^BHB^, or sodium BHB) were s.c. injected 6 h before an i.p. cisplatin injection (Figure 7(a)). Cisplatin injection to control mice (saline-treated group) significantly increased plasma levels of BUN and CRE, the two kidney functional damage biomarkers, compared to mice without cisplatin injection (healthy group) (Figure 7(b,c)). This is consistent with previous studies about the nephrotoxicity of cisplatin [13,36–39]. While the treatment with LMW sodium BHB showed no different effects from saline after cisplatin injection, the BUN and CRE levels of Nano^BHB^-injected mice were not elevated as high as that of the disease control and sodium BHB groups. This suggests the protective effects of Nano^BHB^ against the lost renal function caused by cisplatin toxicity. Periodic acid-Schiff (PAS) staining of kidney tissue showed obvious cisplatin-induced protein casting in both saline- and sodium BHB-treated groups, while such casting was reduced in the Nano^BHB^ group (Figure 7(e,f)). Since these castings represent injury areas in kidney tissue, these results demonstrate that Nano^BHB^ protected the kidney against damage caused by cisplatin injection. Besides, Nano^BHB^ also suppressed the elevated AST levels (a liver function damage biomarker) induced by cisplatin in a better manner than sodium BHB (Figure 7(d)), suggesting that Nano^BHB^ might also possess positive effects in livers. Overall, these results indicate that Nano^BHB^ exhibited better protective effects than LMW BHB and effectively attenuated the renal damage of cisplatin-induced AKI.

(a) Scheme of animal experiments; (b) Blood urea nitrogen, (c) Creatinine, and (d) Aspartate transaminase in plasma of AKI mice 3 days after injections; (e) Presentative images of Periodic Acid−Schiff (PAS) staining of kidney tissue at 20× magnification, and (f) Quantitative measurement of injured area in kidneys from staining images. (Data are displayed as Mean ± SD, *n* = 8-9, *p < 0.05, **p < 0.01, ***p < 0.001,****p < 0.0001, ns: not significant difference with *p* > 0.1)

### 3.6. Possible mechanisms for the effects of Nano^BHB^

**Figure 8.**
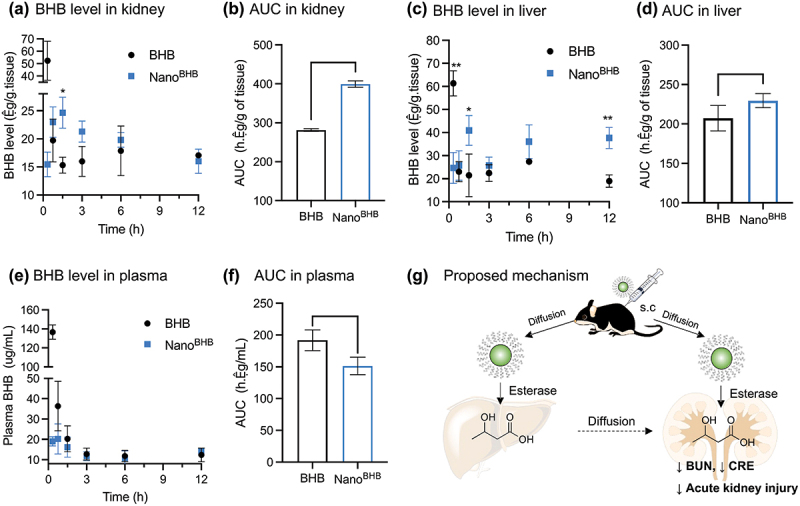
Bioavailability of BHB after Nano^BHB^ injection and proposed mechanism.

We hypothesized that the improved nephroprotective effects of Nano^BHB^ could be related to the extended release of BHB after administration. Pharmacokinetic experiments, especially in kidneys, were conducted to investigate BHB levels after subcutaneous injection of Nano^BHB^ and compare them to those of LMW BHB. As shown in Figure 8(a), the LMW BHB injection immediately increased the kidney’s BHB level and completely disappeared after 1.5 h (half-life time *t*_1/2_ <20 min). Meanwhile, Nano^BHB^ gradually supplied BHB and reached the highest concentration at *t*_max_ = 1.5 h, followed by a sustainable release pattern until 12 h (*t*_1/2_ ≈ 3 h). In addition, the bioavailability of BHB supplied from Nano^BHB^ in kidneys is significantly higher than that of LMW BHB, displayed via the higher AUC of BHB levels in kidneys (Figure 8(b)). It is reported that the kidney has a certain level of esterases [40,41], and small-sized nanoparticles tend to accumulate in kidneys due to blood filtration [42,43]. To note, polymer micelles like Nano^BHB^ are stable even under super-diluted conditions below critical micelle concentration (e.g. within the bloodstream) because of the improved stability by the entanglement of the hydrophobic chains in the core, which is not a thermostable but kinetic control process [44,45]. Because of this kinetic condition, Nano^BHB^ is expected to disintegrate gradually in physiological environments. Therefore, as soon as the accumulated Nano^BHB^ in kidneys starts to disintegrate and exposes the interior poly(D,L-BHB), subsequent hydrolysis by renal enzymes gradually liberates BHB and improves BHB bioavailability in kidneys.

Levels of BHB over time in kidneys (a), liver (c), plasma (e), and corresponding area under the curve analysis (b, d, f); (g) A proposed mechanism for the bioactivities of Nano^BHB^. (Data are displayed as Mean±SD, *n*=3, *p < 0.05, **p < 0.01, ***p < 0.001,****p < 0.0001).

Compared to the LMW BHB, liver BHB levels in the Nano^BHB^-injected mice were also elevated for up to 12 h after injection (Figure 8(c)), resulting in a higher liver AUC (Figure 8d). Since nanoparticles were reported to accumulate in the liver due to the uptaking of mononuclear phagocyte systems [46] and there is a large number of hepatic endogenous esterases [47–49], the disintegration of Nano^BHB^ and enzymatic hydrolysis to continuously release BHB from Nano^BHB^ also occur in the liver. This results in an increase in BHB levels in livers over time, which can diffuse to the whole body and accelerate the increase in BHB levels in kidneys. Although BHB supply from Nano^BHB^ is not robust enough to induce significant changes in plasma BHB levels compared to that from the LMW compound (Figure 8(e,f)), a small but definite level of sustained BHB from Nano^BHB^ is essential to exert protective effects against cisplatin nephrotoxicity in this study (Figure 7). Altogether, we propose a possible explanatory pathway for how Nano^BHB^ works after injection as illustrated in Figure 8(g): Nano^BHB^ releases free BHB in the kidneys and liver, resulting in a sustained increase in renal BHB levels and nephroprotective effects against cisplatin-induced acute kidney injury models.

## 4. Conclusion

In conclusion, we have developed Nano^BHB^, a biocompatible nano-sized formulation in aqueous conditions, which is suitable for both oral administration and injection routes. Nano^BHB^ was designed from amphiphilic block copolymers of mPEG and poly(D,L-BHB), which are hydrolyzed by endogenous enzymes and result in the sustained liberation of BHB. The treatment with Nano^BHB^ showed improved bioavailability of BHB in kidneys and superior nephroprotective effects in the acute kidney injury models compared to that of LMW compounds, suggesting that Nano^BHB^ could be a potential source for sustaining BHB supply in treating AKI.
